# Quantum Mechanical Modeling: A Tool for the Understanding of Enzyme Reactions

**DOI:** 10.3390/biom3030662

**Published:** 2013-09-23

**Authors:** Gábor Náray-Szabó, Julianna Oláh, Balázs Krámos

**Affiliations:** 1 Laboratory of Structural Chemistry and Biology and HAS-ELTE Protein Modeling Group, Eötvös Loránd University, Pázmány Péter St. 1A, Budapest H-1117, Hungary; 2 Department of Inorganic and Analytical Chemistry, Budapest University of Technology and Economics, Gellért tér 4, Budapest H-1111, Hungary; E-Mails: julianna.olah@mail.bme.hu (J.O.); kramosbalazs@ch.bme.hu (B.K.)

**Keywords:** enzyme, reaction, mechanism, model, quantum mechanics, QM/MM

## Abstract

Most enzyme reactions involve formation and cleavage of covalent bonds, while electrostatic effects, as well as dynamics of the active site and surrounding protein regions, may also be crucial. Accordingly, special computational methods are needed to provide an adequate description, which combine quantum mechanics for the reactive region with molecular mechanics and molecular dynamics describing the environment and dynamic effects, respectively. In this review we intend to give an overview to non-specialists on various enzyme models as well as established computational methods and describe applications to some specific cases. For the treatment of various enzyme mechanisms, special approaches are often needed to obtain results, which adequately refer to experimental data. As a result of the spectacular progress in the last two decades, most enzyme reactions can be quite precisely treated by various computational methods.

## 1. Introduction

Enzyme reactions are extremely complicated processes involving directly or indirectly a large number of atoms in the chemical rearrangement of the reacting species. Accordingly, their understanding in atomic details is also very difficult and needs combined application of a variety of experimental and molecular modeling techniques. Rate and binding constants, as well as inhibitory power provide direct information on energetic aspects; however, they are bulk descriptors of the reaction and give information on subtleties only in cases when they can be associated with a single step. A direct method, X-ray diffraction, helps in constructing three-dimensional models at the atomic resolution but does not allow drawing conclusions on energetic aspects of the process. Detailed and precise elucidation of an enzymatic reaction is therefore not possible using present-day experimental methods alone; application of molecular modeling seems to be a must in order to clarify subtle details.

Because of the very large number of atoms involved in the reaction enzymatic processes, in general, cannot be modeled as accurately as interactions between small molecules with a few atoms. Though in some specific cases, by the application of high-level quantum mechanical methods quantitative agreement with experiment could be achieved [1], this is presently not routine. A promising way to obtain a detailed and adequate description of the process is to combine experimental evidence with modeling techniques. The reliability of a model can be checked by comparing calculated and experimental data and if an appropriate agreement is achieved, one may be confident on its validity.

With the spectacular development of computer hardware and software a substantial progress has been made in the development of quantum chemical methods. High-performance models of solvation, as well as combination of *a priori* quantum mechanical and empirical molecular mechanical approaches have become an integral part of commercial molecular modeling program packages. Along with the dramatic increase of the computer performance to price ratio, computational chemistry became suitable even for predictions, and thus it complements experimental findings. It seems that direct mapping between calculated and experimentally observed properties, as well as molecular structures represents a major advantage of quantum chemical modeling since this type of information is very difficult to obtain experimentally. If the observed properties, related to enzyme reactions, can be quantitatively reproduced by calculations we may consider reaction profiles and other calculated parameters to be reliable, too. Agreement between theory and experiment may imply that the reaction mechanism of the studied enzyme can be considered as clarified. 

In this review we give an overview first on models, which can be used for the understanding of an enzyme reaction. Then, up-to-date and popular methods of calculation will be surveyed without laying emphasis on their mathematical background and technical details, rather focusing on their performance. The main part of the paper is written on case studies on some enzyme reactions, either involving special effects or dealing with a widely studied class of processes. First we discuss serine proteases, an important enzyme family for which three-dimensional structures have been available very early [2]. While the key reaction, general base assisted catalysis, is well-known in organic chemistry, a variety of factors contributing to rate enhancement has to be considered, and it seems that by now most of them are understood in detail [3]. Next we treat biological phosphate ester hydrolysis, which is a key step e.g., in the transfer of the phosphoryl group from a phosphate ester or anhydride to a nucleophile [4]. Understanding of this process is closely linked to the elucidation of mechanisms for the corresponding non-enzymatic reactions in solution. A wide variety of experimental data is available; however, it is difficult to summarize indirect conclusions in the absence of explicit molecular models. Accordingly, quantum mechanical calculations provide an important option to obtain a reasonable guess for the reaction energy profile. An interesting process is long-range electron transfer in heme peroxidases*,* its understanding in detail involves special experimental techniques. Therefore, to study enzyme-catalyzed electron transfer reactions we need to perform sophisticated quantum mechanical calculations, classical molecular mechanics alone is not appropriate (cf. Section 4.3). Cytochrome P450 enzymes form a very large superfamily of heme enzymes, their regioselectivity, oxidizing power and reactivity are therefore of outstanding interest, we treat them in Section 4.4. At last we deal with a special case, xylose isomerase catalysis [5], where quantum effects, namely proton tunneling, play a certain role in determining the reaction rate. Tunneling could be relatively precisely reproduced by direct calculations on other enzyme reactions, too.

## 2. Models

**Figure 1:**
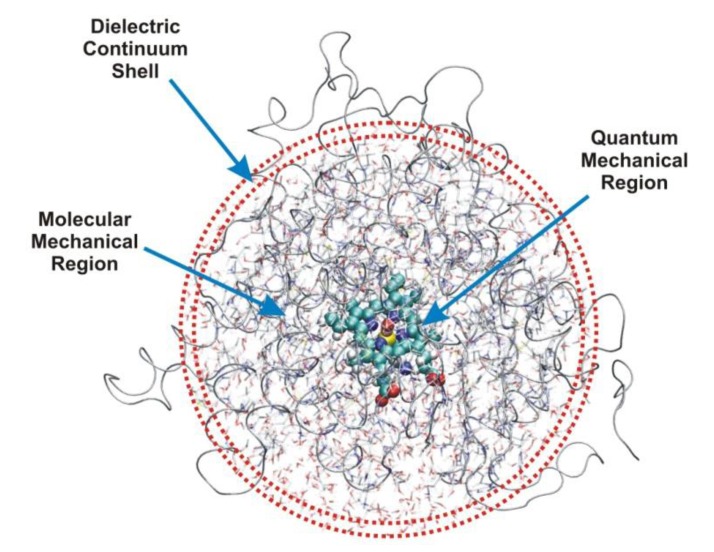
Hierarchical composition of a full enzyme system. Active site or quantum mechanical region (**A**); protein core or molecular mechanical region (**P**); bulk or dielectric continuum shell (**B**) (figure drawn on the basis of the crystal structure of human aromatase, 3EQM).

An enzyme has thousands of atoms, therefore the whole molecule and its transformations cannot be treated by direct quantum mechanical methods. Instead of striving for complete models, including all atoms of the enzyme and its surroundings, the system is better partitioned into various regions, which can be described at various levels of sophistication. The active site (**A**) is embedded in the amino-acid residues of the protein core (**P**) with ionizable surface, eventually buried, side chains and the whole protein is dissolved in the bulk (**B**). For most enzymes studied, this latter contains, beside water molecules, counter ions, partly or fully shielding the electrostatic field of the positively or negatively charged side chains. However, in some specific cases, the bulk is not necessarily aqueous, it can be formed by e.g., the atoms of a membrane, where the enzyme is located. The above three regions, schematically depicted in Figure 1, can be calculated at different levels of sophistication.

For most enzymes **A** includes the catalytic machinery, *i.e.*, key amino-acid side chains, one or more water molecules and substrate, these fragments are directly involved in catalysis. The minimum number of non-hydrogen atoms in this model may range from 10 to 200, which should be extended by a further number of atoms belonging to essential prosthetic groups, if present. Up-to-date quantum mechanical methods are available even for the high-level treatment of such systems, even if these contain one or more third or higher row atoms, e.g., sulfur, phosphorus or some transition metal. Special electronic effects, like excitation or transfer can be accounted for using sophisticated computational methods, which make use of high-performance software and hardware. The basic reason, why quantum mechanics is indispensable for the adequate description of these models, is that bond fission and formation taking place during the vast majority of enzymatic processes needs such an exact treatment. We may obtain structural parameters for **A** mainly from the Protein Data Bank [6], in some specific cases from other sources. If geometry optimization is necessary, atoms at the boundary of **A** must be fixed in order to avoid artificial distortions from the experimental, chemically relevant structure, which may essentially influence final results and lead to artifacts in the modeling procedure. The atoms of the active site must be kept within the geometric frame of the protein core, which is absent from **A**, therefore sometimes spurious effects may arise as a result of geometry optimization potentially leading to lethal distortion of the active-site structure. In a concrete model covalent bonds linking side chains or other groups of the active site to atoms in **P** must be split, while the resulting dangling bonds can be saturated by hydrogen atoms, eventually methyl groups. This is quite feasible for apolar single C**–**C bonds, but for polar links, like C–N or C–O replacement of an atom with another, for which the electronegativity is essentially different from that of hydrogen, it causes spurious charge accumulation at the boundary. Similar effects may occur if multiple bonds are cut. Most preferably, the active site should be constructed by cutting C^α^–C^β^ bonds of the amino-acid residues. A further problem is that hydrogen atoms, used for the saturation of dangling bonds emerging after cutting, may be in steric conflict with some atoms of **P** and this may even lead to convergence problems in quantum mechanical calculations as well as spurious terms in the energy expression within a force field applied in the molecular mechanical calculation. In case of some electron transfer reactions it is very difficult to define the active site atoms appropriately, since localization of an unpaired electron may be uncertain. In these cases, results are very sensitive to the definition of the model, adding or dropping one or two atoms may considerably influence, e.g., the calculated spin density distribution. In such cases, the gradual extension of the size of the model may bring certain saturation in calculated sensitive properties, like charge or spin distribution. In a series of calculations on models with gradually increasing size, the smallest model for which these properties do not change, as compared to previous one, may be appropriate. 

Enzymatic mechanisms cannot be understood quantitatively on the basis of active-site models alone. Even in cases, like phosphoryl transfer (cf. Section 4.2), where the basic reaction step seems to be determined by covalent bond fission and formation within the active site, electrostatic, steric and hydration effects influence the formation, structure and protonation state of the active site, which may have some or even basic importance. At least two important phenomena, electrostatics and protein fluctuation cannot be reduced solely to the active site; distant protein residues may and quite often do play a role. In case of electrostatics incorporation of atomic monopoles in the model provides often quite good results; however, sophisticated non-quantum mechanical methods are available for the consideration of such effects. Atomic monopoles can be treated as transferable from one protein to the other; this means, however, that mutual polarization between the active site and protein residues is neglected. This approach is quite feasible; but even semi-quantitative agreement with experiment is rarely achieved. Backbone and side-chain fluctuation can be treated on the basis of molecular dynamics (see Section 3); restriction of the number of protein atoms in the model is allowed only, if local effects (e.g., separated side-chain fluctuation) are investigated.

In most cases the overwhelming majority of the bulk, **B**, is water; however, dissolved inorganic ions and eventually other components are quite important when estimating its effect on the reaction process. The dielectric constant of water is large; therefore the effect of **B** on electrostatic factors, influencing the reaction, may be quite important. In a precise model it is not sufficient to consider only **A** and **P**, the influence of **B** must also be estimated some way. Water molecules may influence the outcome of the enzyme reaction by three different ways. One or more molecules may act as proton donor or proton acceptor during reaction, these have to be explicitly included in **A**. Structural water molecules bind quite strongly to the protein core or the surface, because of their reduced mobility they have to be explicitly included in **P**. Locations can be obtained from X-ray diffraction studies, for which results are deposited in the Protein Data Bank. Like for other atoms, belonging to the protein core, electrostatic effect of structural water molecules can be considered by including appropriate point charges in the Hamiltonian. In case of a molecular dynamics study **P** must include all structural water molecules explicitly. The most complicated type of water in biological systems is bulk, for which a manifold of models with varying adequacy is available. It is possible to model the bulk by a finite set of point charges, however, their electrostatic effect converges very slowly, thus a very large number of molecules has to be included. An implicit way is to use an empirically selected dielectric constant in the force field. Because of the strong effect, *i.e.*, the large dielectric constant of bulk water on protein electrostatics, energy differences for various intermediates and transition states of the reaction are reduced to a quite large extent as compared to the corresponding reaction in the gas phase. Accordingly, great care is needed when comparing quantum mechanically computed values to experimental ones. The bulk may contain small inorganic ions (e.g., Na^+^, K^+^, Ca^2+^, Cl^–^, HO^–^), which partly shield the charge of surface side chains, their concentration is characterized by the ionic strength. Counter ions are not fixed; rather they form a loose distribution of charges. Their effect can be best simulated by high-performance methods like the Poisson-Boltzmann equation, to be discussed in the next section. In some cases when the enzyme reaction is diffusion-controlled the process can be described by Brownian dynamics simulation techniques. These are developed to estimate the rate at which the reactant molecules would collide with the active site in the appropriate orientation [7]. 

The role of protein dynamics in enzyme catalysis is a controversial question in computational enzymology. Some claim that protein dynamics are essential in understanding enzymatic processes, while others state that dynamics is not an important contribution to catalysis. However computer simulations demonstrate the importance of structural fluctuations and the need to include them in the modeling of certain enzyme reactions [8,9]. In some cases, e.g., in processes with significant changes in solvation it is even impossible to correctly predict activation energies if the dynamics of the protein and its active site is not considered in the calculation. In other reactions fluctuations have only a slight effect on the reaction path (see trypsin in Section 4.1).

It is often supposed that entropy effects play an important role in enzyme catalysis by fixing the reaction partners in the proper orientation and thus reducing translational and rotational entropy in the transition state. This means that an “entropy trap” may contribute very much to rate acceleration. However, these effects may be smaller than anticipated since enzyme molecules are quite flexible. For example in case of serine proteases entropic contribution to rate acceleration is relatively small, rather preorganization of the active site to ensure maximum interaction between reacting partners is the basis of rate enhancement [10]. As we will show in Section 4.1 electrostatic stabilization of the active-site complex plays here a prominent role, while in other cases the liberation of water molecules from the active site into bulk solvent may be also crucial. 

## 3. Methods

In this section we give an overview on some popular methods for the quantum mechanical treatment of enzyme reactions, more details can be found in recent reviews [11,12]. It must be stressed that presently available methods cannot be considered as black-box applications, those who apply them must have some experience in judging the reliability of some of their special features. Otherwise, potential artefacts may appear, which become especially dangerous, if remain hidden. For example, in case of an ill-defined model of the boundary between **A** and **P** iteration, needed to reach the quantum mechanical result, may diverge. This is easily detectable, however, if the iteration converges to a false value, the mistake may remain hidden.

Depending on the size of the active site (**A**) and the required accuracy various quantum mechanical methods can be selected for a calculation on the restricted model, **A**, which contains some dozens of atoms. Several software packages are available for the application of these methods at higher or lower accuracy, like the latest version of the GAUSSIAN program package [13], which incorporates several specific applications. In case of enzyme reactions it is a very appealing feature that it allows to compute the reaction path and the activation energy and to determine the structure of reactants and products, which are connected by a given transition structure. Basically, either the *ab initio* molecular orbital [14] or the density functional [15] method can be applied, as far as the size of **A** allows it. If the size of **A** allows, electron correlation can be accounted for in the calculation, which is highly recommended if a covalent bond is formed or cleaved during the enzymatic process. The quantum chemical treatment of transition metal atoms, which are in a number of enzymes essential parts of **A**, is a notoriously difficult task. The metal-ligand interactions are often highly directional and the selection of the appropriate quantum mechanical method is not always straightforward. The problem is even more difficult if the system may exist in several energetically close-lying spin states which are all characterized by different coordination numbers and local geometries. If **A** is very large with more than 200 non-hydrogen atoms, semiempirical methods can be applied which may provide a rough description of the mechanism and pave the way toward a more precise treatment. 

Like in case of partitioning of the whole enzyme system into active site, protein core and surrounding bulk, a layering of the quantum region is also possible. It is the ONIOM method, available within the Gaussian package that allows this [16]. The molecular system under investigation is partitioned into three layers, which are described at successively more accurate levels. The innermost one, where bond formation and breaking takes place, is treated with the most accurate method, referring to electron correlation, if necessary. The outermost layer corresponds to **P** + **B** and is treated with molecular mechanics, a semi-empirical or an *ab initio*, small basis set Hartree-Fock method. The middle layer, which is sometimes dropped from the model, is treated with a method of intermediate accuracy between those applied to the high and low-level layers. It is necessary if electronic effects play a role in **P**, which cannot be treated by any molecular force field.

If the size of **P** is too large for a quantum mechanical method molecular mechanics can be applied for the calculation of various hypothetical or real structures and their energies [17]. In this approximation molecular systems are considered in the frame of the classic Newtonian mechanics approach. The energy is expressed as a sum of bond stretching, bending, torsional, non-bonding and cross terms. These contributions are estimated using mathematically very simple expressions; therefore, calculation of energy changes is very fast, several orders of magnitude faster than in the case of quantum mechanical methods. In earlier versions, the parameters of these simple expressions are fitted either to experimentally determined structural quantities, like bond lengths, bond angles, equilibrium torsional angles, vibration spectra, thermochemical and other data. Up-to-date methods of parameterization refer to high-precision quantum mechanical calculations, which is a better approach since direct correspondence between calculated and estimated parameters can be achieved. It is supposed that parameters are transferable within a relatively large set of molecules, which are constructed of similar fragments. Bonded parameters can be approximated quite precisely; bond lengths and angles, as calculated by molecular mechanics, reproduce experimental values very precisely. Determination of non-bonding parameters often means a problem. Van der Waals parameters can be obtained relatively accurately; however, definition and derivation of atomic point charges is not straightforward, even in modern force fields. The basic problem is that the charge distribution of an atom is not perfectly defined by the simple Coulomb monopole expression, dipole and higher moments. Furthermore, polarization and electron delocalization should be considered, too. In some cases the two-atom term additivity does not hold perfectly, three-body and higher interactions may be essential.

For a wide class of biomolecules, including proteins and nucleic acids the AMBER parameterization and software package became very popular. Since its first publication in 1981 it has been further developed several times until it reached its present version [18]. The original AMBER force field and program package was used for the search for energy minima of separate molecular systems in the gas phase. The current versions are aimed more at the simulation of biomolecules in water solution. Both explicit and implicit models for water as well as for some organic solvents are accounted for. Beside AMBER, several other force fields are available in the literature. For example, GROMOS force fields do not consider aliphatic hydrogen atoms. In contrast to most other ones, this parameterization aims primarily at reproducing the free enthalpies of hydration and apolar solvation for a range of organic compounds [19]. A versatile software GROMACS was developed at the University of Groningen, starting twenty years ago [20]. This is a fast program for molecular dynamics simulation, which does not have a force field of its own, but is compatible with several parameterizations. It was developed and optimized for use on PC-clusters. The CHARMM software is also widely used; it has been developed with focus on biomolecules, like proteins. With its newest parameter set it can be used with various energy functions and models, e.g., combined quantum mechanical-molecular mechanical methods, all-atom classical potentials, explicit and implicit solvent models, as well as various boundary conditions [21]. The OPLS force field is also often applied to elucidate enzyme mechanisms [22].

For the treatment of time-dependent phenomena molecular dynamics methods can be used [23]. The term molecular dynamics (MD) typically refers to the propagation of atoms or groups combining several atoms according to the laws of classical mechanics. The forces acting on the particles are calculated only at discrete points along the trajectory. This method allows computer simulation of local or global molecular movements. The atoms of a molecular system are allowed to interact and the trajectories describing their movement can be calculated by solving Newton’s equations of motion. Forces between the particles and the potential energy of the system are obtained by expressions from molecular mechanics. For a large number of atoms within most systems investigated, it is impossible to solve these equations analytically; numerical methods are needed. Long simulations are often mathematically ill-conditioned thus errors may accumulate, which can be minimized using appropriate algorithms. Molecular dynamics simulations may be used to determine macroscopic thermodynamic properties by calculating statistical averages. 

If quantum effects play a substantive role in the enzymatic process, the *ab initio* molecular dynamics approach can be applied [24]. In this method the quantum mechanical effect of the electrons is included in the calculation of the energy and forces for the classical motion of the atomic nuclei, explicitly including the electrons as active degrees of freedom in the calculation. This is a first principles molecular dynamics approach, which usually employs periodic boundary conditions, plane-wave basis sets, and density functional theory. Application of this method ranges from the thermodynamics of solids and liquids to the study of chemical reactions in solution and quantum mechanical design of enzymes [25]. Referring to the present-day computer capacity, the size of the system studied is limited to about 250 atoms.

Application of classical force fields to enzyme reactions is problematic if covalent bonds are formed or broken during the process. In such cases quantum mechanical (QM) and molecular mechanical (MM) approaches are combined in a variety of hybrid, quantum classical methods denoted by the acronym QM/MM [11,12]. The active site, **A**, is treated by a quantum mechanical method, while its environment, **P** + **B**, can be described by molecular mechanics. Both Hartee-Fock and density functional methods can be applied in various QM/MM schemes; the latter allows us to include dispersion effects, which is an important feature. The basic assumption in the QM/MM approach is that the system can be partitioned in four regions; accordingly, the Hamiltonian becomes a sum of four terms. Two of them are the QM and MM terms, while the interaction between them (*i.e.*, interaction between **A** and **P** + **B** and their mutual polarization), as well as the boundary between QM and MM regions are treated separately. In most versions polarization of **P** by **A** (the response) is neglected, thus the mathematical procedure becomes much simpler. The boundary, introduced to mimic the rest of the system, is formed of saturated dangling bonds, which remain after cutting the quantum region out of its environment. Several boundary atom schemes have been developed to avoid the artifacts due to them. Thus, the saturation may be done by hydrogen atoms, dummy centers or special, strictly localized molecular orbitals [26]. A boundary center appears as a normal atom in the molecular mechanical calculation.

In order to provide an accurate thermodynamic description of reaction processes in solution and in enzymes free energy perturbation schemes can be combined with QM/MM calculations [27]. The smooth connection between the QM and MM regions in the boundary is crucial to avoid instabilities during the iterative optimization as well as in the free energy calculations. The basic idea is to sample only the molecular mechanics degrees of freedom while the quantum mechanically related atoms are kept fixed. This allows a significant reduction of the computational effort. Warshel and co-workers proposed that the sampling is based on the empirical valence bond (EVB) scheme [28,29], where parameters are fitted to *ab initio* data [30].

A variety of enzyme processes is determined, or at least influenced, by the electrostatic field of the protein core and the bulk. Quite often, the polarity of transition-state structures is different from those of the initial or final ones, which means that their electrostatic interaction with the environment is also different. Protonation and deprotonation, almost always determined by electrostatics, are also important part of the enzymatic reaction process. Thus, for a precise description of the energetic aspects of the reaction, considering the electrostatic effect of the environment on the various steps of the reaction is crucial. One of the most effective and popular methods for the adequate estimation of protein electrostatics is the solution of the Poisson-Boltzmann equation for the whole system containing **A**, **P** and **B**, as well [31]. The method is based on the Poisson equation, which relates the spatial variation of the electrostatic potential as a function of the charge density and the dielectric polarizability. In case of a uniform polarizability, represented by a single dielectric constant, and a point charge distribution the Poisson equation is reduced to the simple Coulomb’s law. If the polarizability is not uniform, the dielectric constant is a function of spatial co-ordinates. It is possible to consider the effect of ions dissolved in the bulk via representing the mobile ions by a mean-field approximation. The ionic concentration is determined by the Boltzmann equation and replacing the charge density in the Poisson equation by the sum of densities due to dissolved ions and the protein a combination, the Poisson-Boltzmann equation is obtained. This equation can be applied to systems including **A**, **P** and **B**, which is described by a non-uniform dielectric constant. If the protein does not accumulate a high amount of charges, the equation can be linearized allowing a numeric solution. Advanced finite difference methods can be applied with success and a commercial software is available, which can be applied even by the non-specialist [32].

The combined effect of the **P** and **B** regions is recently more and more frequently treated by the Polarizable Continuum Model (PCM) developed by Tomasi and coworkers [33]. This calculates the molecular free energy in solution as the sum of electrostatic and dispersion-repulsion contributions extended by the cavitation energy, which is needed to form the solvent cavity embedding the solute. The electrostatic energy is calculated from point charges located on the molecular surface, which is constructed from spheres representing non-hydrogen atoms with appropriate van der Waals-radii. The dispersion-repulsion contribution is calculated using the solvent accessible surface. The PCM is clearly less precise than the sophisticated Poisson-Boltzmann method; however, it allows much faster calculations. Its use may be amended in cases where the protein core surrounding the active site can be considered as uniform to at least a certain extent. In cases if **P** consists of several ionizable side chains and ionic strength effects in **B** are thought to be important, the Poisson-Boltzmann method is recommended.

## 4. Case studies

### 4.1. Electrostatic Catalysis: Serine Proteases

Over one third of known proteolytic enzymes are serine proteases [34]. They are found both in prokaryotes as well as in eukaryotes and cleave covalent peptide bonds of proteins. At the molecular level this is a very important event in case of several life processes. For example, serine proteases digest food for bacteria and help viruses to infect cells. These enzymes co-ordinate various physiological functions in humans, e.g., digestion, immune response, blood coagulation as well as reproduction. They were given this name because their active site includes a highly reactive serine side chain, which is crucial for catalytic activity and is part of a Ser-His-Asp triad, which is buried in the protein core. The mechanism of action is identical in the various members of the serine protease family; they differ only in substrate specificity. The spatial arrangement of amino acids in the triad is optimized for catalysis by convergent evolution at the molecular level [35].

**Figure 2:**
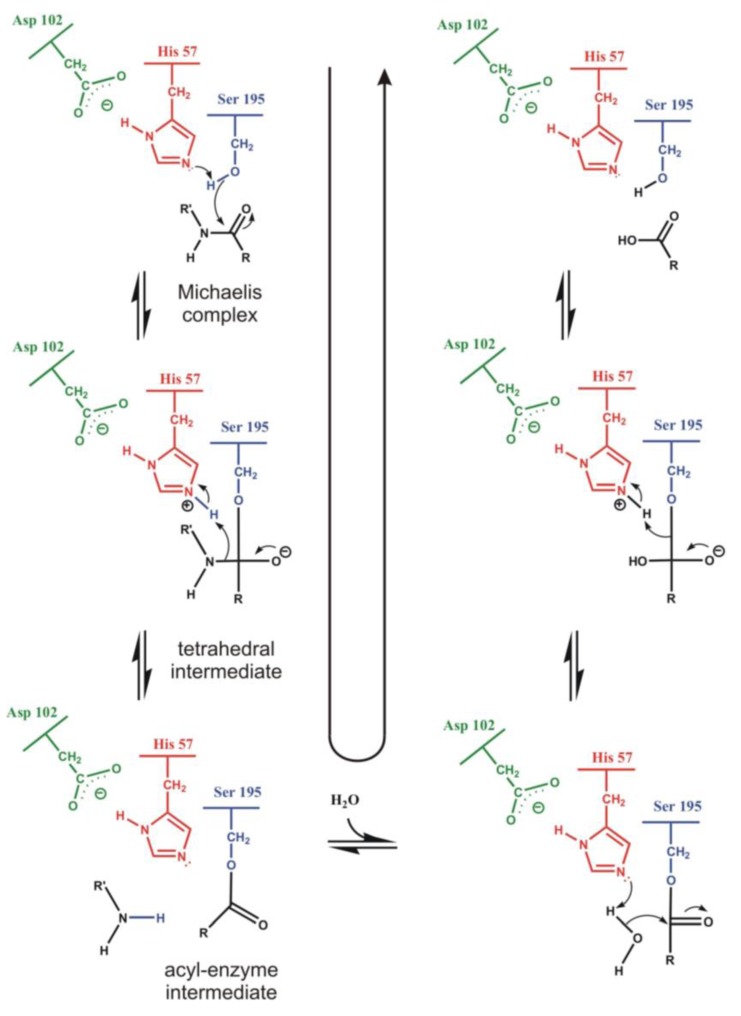
Reaction steps during serine protease catalyzed cleavage of the peptide bond (**left**). The acyl-enzyme intermediate hydrolyses via the reverse route (**right**).

Serine protease-catalyzed hydrolysis of the peptide bond is a reaction involving a tetrahedral intermediate (see Figure 2). During catalysis a nucleophilic attack by the serine hydroxyl group on the carbonyl carbon atom of the substrate is helped by the histidine imidazole group as a general base. This leads to formation of a tetrahedral intermediate and an imidazolium ion. In a subsequent step the tetrahedral intermediate is broken down by general acid catalysis to an acyl-enzyme, an imidazole base and an amine product. During the acylation step, the imidazole group transfers the proton of the serine hydroxyl to the amine leaving group. The acyl-enzyme is then deacylated through the reverse reaction pathway of acylation, but in the second addition-elimination reaction a water molecule instead of the serine residue is the attacking nucleophile.

**Figure 3:**
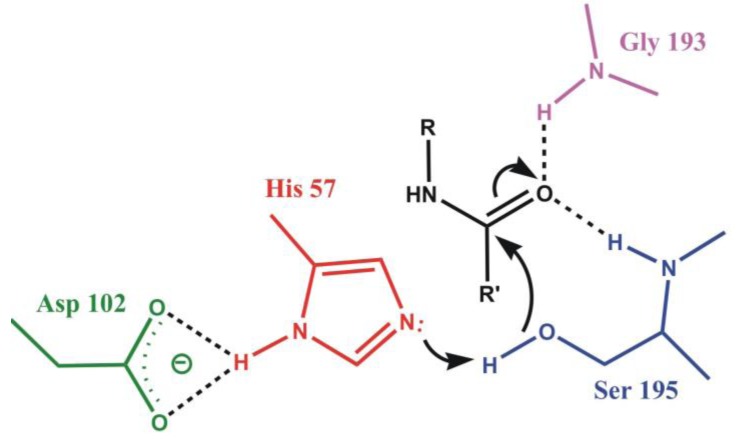
The oxyanion hole stabilizing the tetrahedral intermediate in α-chymotrypsin. Backbone amide NH groups of Gly193 and Ser195 form hydrogen bonds with the amide oxygen atom of the substrate.

Crucial step of the reaction is the formation of the tetrahedral intermediate, facilitated by the surrounding oxyanion hole, stabilizing the intermediate by hydrogen bonds (see Figure 3). Backbone amide groups form hydrogen bonds with the strongly polarized oxygen atom, keeping the carbonyl bond in the right position for a nucleophilic attack and stabilizing the intermediate structure. Formation of the oxyanion hole is a general feature of serine proteases and it is precisely tailored for the oxygen atom. Thionoester substrates, containing sulfur with a somewhat larger atomic radius than the oxygen atom, are not hydrolyzed by chymotrypsin and subtilisin, although they do not differ significantly from esters in reactivity [36]. The contribution of the oxyanion binding site to the catalytic rate acceleration was estimated by replacing the hydrogen-bond donor Asn155 of subtilisin with a neutral glycine residue. This lowered *k*_cat_ for a specific substrate by a factor of 150 to 300, however, did not alter the Michaelis constant, *K*_M_ at all. Thus, it can be concluded that the binding site contributes only to transition-state stabilization and leaves the binding power unaffected [37].

X-ray diffraction studies have shown that catalysis is assisted by a hydrogen-bonded triad of amino-acid side chains. A buried aspartate is linked to the imidazole moiety of histidine, which binds to the catalytic serine hydroxyl group (see Figure 2.) [38]. The geometric relation of the Asp, His and Ser side chains allows assuming that histidine serves for transferring the proton from Ser to Asp in a mechanism called charge relay. However, since proton transfer from the highly basic serine hydroxyl group to the acidic carboxylate side chain of aspartate is unlikely, it was supposed that the role of the buried aspartate is the stabilization of the ion-pair formed by the positive imidazoliun ion and the negatively charged-tetrahedral intermediate [39]. Nuclear magnetic resonance [40] and neutron diffraction [41] studies have confirmed that it is the imidazole and not the aspartate that is protonated. 

The stabilizing role of the buried aspartate is also supported by site-directed mutagenesis. Replacement of Asp102 of trypsin with a neutral Ala residue results in a reduction of four orders of magnitude in the rate constant. It is evident from the three-dimensional structure of the mutant enzyme that the catalytic His57 is unable to accept proton from Ser195 [42]. Studies on the D641A mutant of prolyl oligopeptidase, belonging to another family of serine proteases, also indicate the importance of the negative charge of the buried aspartate [43]. It is thus quite clear that it electrostatically stabilizes the ion pair formed in the transition state. This has been shown by empirical valence bond free energy calculations, too [44]. It has been found that in trypsin and subtilisin the buried aspartate contributes to the stabilization of the transition state by 26 (25) and 17 (25) kJ/mol, respectively (experimental values are in parentheses). These calculations clarified the role of the buried aspartate side chain, which is electrostatic stabilization of the ion pair formed between the positive imidazolium side chain and the negative tetrahedral intermediate rather than proton transfer to its carboxylate.

It is interesting to note that mutation of all three members of the catalytic triad to alanine does not reduce activity to zero. This implies that the catalytic triad is not the sole source of the enzyme activity since even in its absence the mutant enzyme hydrolyzes substrates three orders of magnitude faster relative to the reaction in water. This remaining activity may arise from the contribution of stabilization by the preorganized binding pocket and the oxyanion hole. Calculations by Warshel and coworkers provide ample evidence for the crucial role of electrostatic effects due to the preorganization of active sites, which strongly stabilize the transition states of enzymatic reactions [45,46]. Such a preorganization could happen via convergent evolution organizing the hydrogen-bond network around the catalytic triad in such a way that it stabilizes the (- + -) charge distribution. Poisson-Boltzmann electrostatic calculations provided relative stabilization energies, calculated as the electrostatic interaction energy of atoms of the catalytic triad with the protein environment. These numbers reflect changes in relative experimental enzyme activities, proportional to log *k*_cat_/*k*_M_ for the same substrate, correctly (cf. Table 1) [47].

**Table 1:** Calculated relative electrostatic interaction energies, *vs.* relative experimental enzyme activities (kJ/mol) of the catalytic triad in some serine proteases.

Enzyme	Calculated	Experimental
subtilisin Carlsberg	−102.6	−36.5
α-chymotrypsin	−71.9	−34.2
subtilisin NOVO	−62.1	−31.6
β-trypsin	−40.7	−26.7
α-lytic protease	9.3	0.3

On the basis of the above and other calculations we may say that enzymes, acting as a “supersolvent”, can strongly stabilize polar structures, like ion pairs or the charge distribution located in their active sites. Stabilization in enzymes is stronger than in water, since preorganized and fixed protein dipoles are almost optimally oriented at the active site. The enzyme, therefore, provides a preoriented environment that stabilizes the transition state of the reaction. In contrary, water dipoles are distributed randomly and have to reorient in order to stabilize transition states. Reduction of the preorganization free energy in the enzyme is due to its folding into its final configuration, which precedes the catalytic process and takes place independently, during protein synthesis. 

Even if we have diverse experimental evidence for the elucidation of the mechanism of action, direct information, especially on energetic aspects, can be obtained only from molecular modeling. Results should be in line with experimental observation, furthermore, providing subtle details, which cannot be obtained from experiments. For serine proteases, the crucial role of protein electrostatics in enzymatic rate acceleration can be discussed in appropriate detail only by calculation [48]. Early quantum mechanical approaches were based on gas-phase models containing the catalytic triad and the minimum-size substrate [49]. Such models could be extended by consideration of protein electrostatic effects on the reaction process [50]. It was shown by *ab initio* calculations that the electrostatic interaction originating from the hydrogen bond network involving residues around the buried aspartate plays a significant role in lowering the barrier height of the proton transfer. Later on, the basic statements of the above calculations were supported by further studies. Ishida and Kato [51] performed calculations on the entire reaction path of the acylation step catalyzed by trypsin using *ab initio* QM/MM methods combined with molecular dynamics. It was found that the rate-determining step is the formation of the tetrahedral intermediate, while breakdown of the intermediate has a small energy barrier. Their calculated activation free energy is 75 kJ/mol. It was shown that the proton transfer from Ser to His and the nucleophilic attack of Ser to the carbonyl carbon of the scissile bond are concerted. The most important catalytic factor of stabilizing the tetrahedral intermediate is the electrostatic interaction between the active site and the protein core. Topf and Richards [52] studied the deacylation step of serine protease catalysis using extended quantum mechanical calculations on a model containing all protein atoms and the bulk as a continuum. The calculations show that in the enzyme the tetrahedral intermediate is a relatively stable species lying 30 kJ/mol lower in energy than the transition state. The stabilization is mainly due to the electrostatic effect provided by the oxyanion hole and the buried aspartate. The rate-determining step of the reaction is proton transfer from the attacking water molecule to the histidine side chain; the activation energy is about 92 kJ/mol, 21 kJ/mol, less than for the reference reaction in water, and close to the value obtained by Ishida and Kato. 

**Figure 4:**
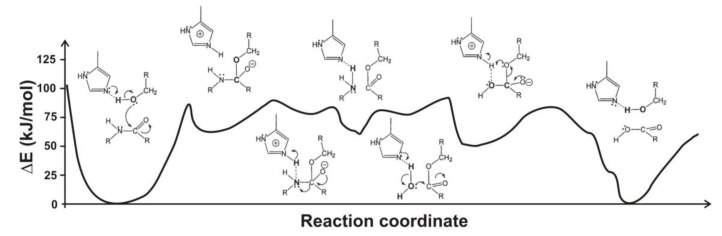
Reaction energy profile of the chymotrypsin-catalyzed cleavage of a model substrate (after Hudáky and Perczel [53]). Note that the tetrahedral intermediates as well as the acyl enzyme represent local minima.

Hudáky and Perczel [53] built a model of the catalytic triad of chymotrypsin containing 18 amino acid residues of the enzyme and its substrate. They found that this model forms a molecule ensemble, which is stable in itself. They obtained an activation barrier of 85 kJ/mol, just between those obtained by the above two groups. Calculations allowed locating elements of the catalytic machinery, which are responsible for the stabilization of the transition state. Most important of these are the catalytic aspartate (by 27 kJ/mol) and the oxyanion hole (by 57 kJ/mol). The full reaction path is displayed in Figure 4. The acylation mechanism of trypsin and its complete free energy reaction profile have been determined also by Born-Oppenheimer *ab initio* QM/MM molecular dynamics simulations with umbrella sampling [54].

### 4.2. Phosphoryl Transfer

Phosphoryl transfer belongs to the most important molecular reactions in living systems. These are involved in DNA replication, signal transduction, metabolism and transcription, furthermore they ensure the production of chemical energy, which is required as a driving force for important processes within living cells [4]. Phosphoryl transfer may be catalyzed by kinases, mutases, phosphatases or endonucleases. Along such reactions enormous, even as large as 10^27^-fold, rate acceleration is observed as compared to the corresponding non-enzymatic process. Despite their central importance, because of the complicated structure of the catalyst, no final information is available about the details of the process although intensive research has been conducted since decades. 

**Figure 5:**
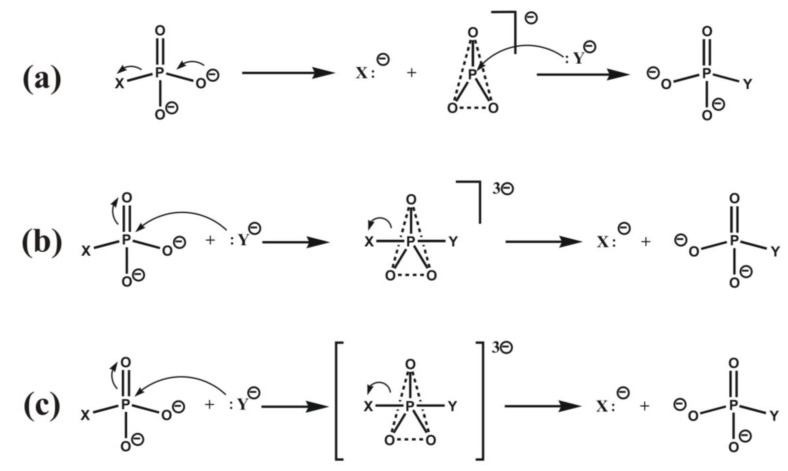
Reaction paths for phosphoryl transfer reactions. (**a**) Dissociative (top), (**b**) associative (middle), (**c**) *S*_N_2-type (bottom) mechanism. Square brackets represent the transition state.

Three different types of reaction paths can be considered for enzymatic and non-enzymatic phosphoryl transfer reactions, these are outlined in Figure 5. In the dissociative mechanism (Figure 5, top) a stepwise displacement takes place via a solvated planar metaphosphate intermediate, while in the associative mechanism (Figure 5, bottom) the reaction proceeds via a trigonal bipyramidal phosphorane intermediate. In both cases the intermediates are locally stable; they represent a local minimum on the reaction energy path. In contrast, in case of the S_N_2-type concerted displacement the pentacoordinated phosphorane structure represents a transition state represented by a maximum on the reaction energy profile.

According to Florián and Warshel [55] both associative and dissociative mechanisms may be operative in the aqueous phase. On the other hand, as stated by Herschlag *et al*. [4] for phosphate monoesters considerable experimental evidence supports a concerted reaction mechanism, where no stable intermediate, rather a transition state can be located on the reaction path. Furthermore, they state that enzymatic catalysis generally does not influence the structure of transition states as compared to those in solution. While this might be true in some cases, we found on the basis of density functional calculations on a model of HIV integrase that metal ions, located at the active site, as well as the electrostatic effect of the protein core considerably reduces the activation energy [56]. The high (295 kJ/mol) reaction barrier in the gas-phase reduces to less than the half, 141 kJ/mol, within the enzyme. This means that the protein environment has an important impact on the reaction, which is partly due to the fact that electrostatic repulsion is reduced in the active site due to a diffuse charge distribution. The nucleophile is only partially charged in the transition state. A further effect is that of the doubly positively charged magnesium ion at the active site, which reduces the barrier height by 42 kJ/mol. A similar result using the density functional theory was obtained for the hydrolysis of guanosine triphosphate [57]. Accordingly, combined geometric and electrostatic effects may play an important role in rate acceleration, like in case of hydrolytic reactions catalyzed by serine proteases (see Section 4.1).

While several experimental arguments can be mentioned strengthening the hypothesis that the concerted mechanism may be operative in enzymes, two different X-ray studies seemed to support the associative mechanism, *i.e.*, the formation of a relatively stable phosphorane intermediate during the catalytic process. Lahiri and coworkers thought to locate such a pentacovalent structure (an XPO3Y moiety) in the phosphoryl transfer reaction catalyzed by *β*-phosphoglucomutase [58]. However, their results have been questioned by Blackburn *et al*. [59] who suggested that this structure represents an MgF_3_^–^ transition-state analogue, which is hydrogen-bonded to ligands at the binding site. Subsequent evidence from ^19^F and ^31^P nuclear magnetic resonance, further X-ray experiments, and kinetic data [60] as well as quantum mechanical calculations [61,62] provided strong support for this latter assignment. A second example of a potentially trapped high-energy intermediate has been published by Barabás *et al*. [63]. Crystal structure of the complex with a special substrate, *α*,*β*-imino-dUTP, was determined. In this case the catalytic process slowed down considerably; therefore the reaction path could be followed by localizing snapshot structures. A relatively stable intermediate was thought to be trapped, for which the crystal structure is available. However, the resolution of the active site structure was not enough to present a convincing argument, so the final proof that the associative mechanism is operative in these enzymes is not yet available.

**Figure 6:**
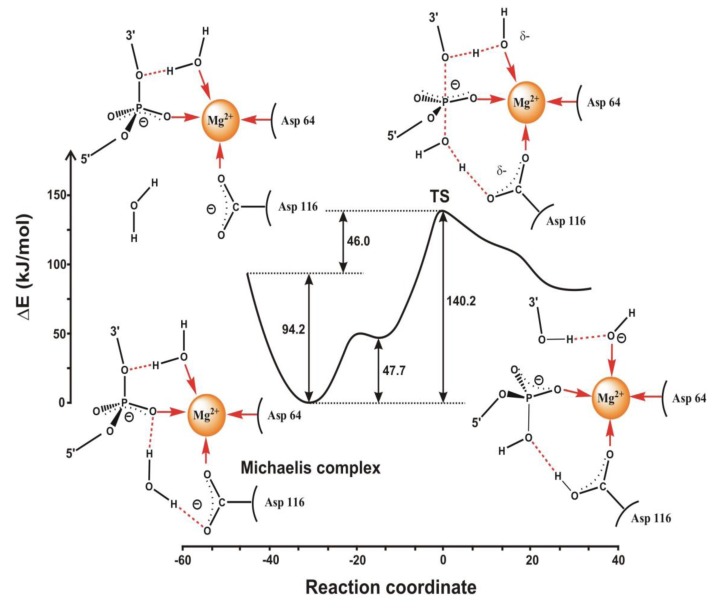
Quantum mechanically calculated reaction profile for the phosphoryl transfer reaction catalyzed by HIV integrase (figure drawn on the basis of Ref. 56).

Quantum mechanical studies on various enzymes catalyzing phosphoryl transfer support different mechanisms. Our early calculations for HIV integrase [56] indicate that an S_N_2-type mechanism may be effective. We calculated the reaction path carefully and found a very shallow minimum, rather a shoulder on the reactant side (cf. Figure 6). This means that if environmental effects, mainly electrostatics, influences the stability of the transition-state complex, under certain circumstances a locally stable phosphorane intermediate can be formed. Thus, the associative reaction route may be realized. Studies for the DNA repair enzyme endonuclease IV [64] as well as for cyclin-dependent kinase [65,66], DNA polymerase [67] and phosphodiesterase [68] support S_N_2-type concerted mechanisms, too. A recent study stresses that a water mediated and substrate assisted mechanism is followed in DNA polymerase, and provides an asymmetric intermediate, which is stabilized by nearby amino-acid residues [69]. This intermediate transforms to the product via a pentacovalent phosphorane transition state, which remains stable for over a nanosecond of MD simulation. This study provides further support for the concerted mechanism because the lifetime of a true phosphorane intermediate should be much longer. 

**Figure 7:**
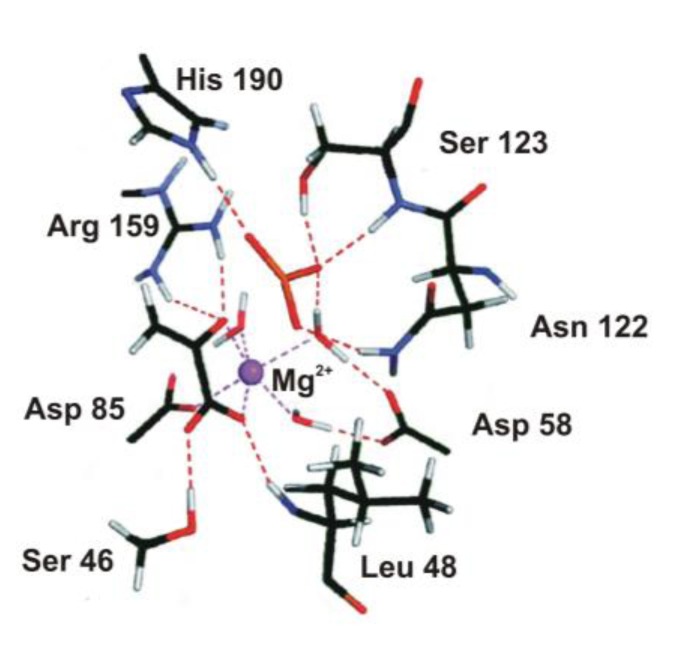
Molecular graphics model of the transition state in the reaction catalyzed by phoshoenol-pyruvate (PEP) mutase (on the basis of Figure 3 by Xu and Guo [70]). Note the planar metaphosphate intermediate stabilized by hydrogen bonds to amino-acid residues of the active site.

*Ab initio* QM/MM studies on phoshoenol-pyruvate (PEP) mutase suggest that the catalytic reaction follows a concerted mechanism with a planar trigonal transition state representing metaphosphate (see Figure 7) [70]. This mechanism is consistent with the failure in detecting the putative intermediate in rapid quench experiments and is in line with the statement of Herschlag and coworkers, who call for a concerted reaction mechanism in case of phosphate monoesters [4]. It is interesting to note that the transition state is planar trigonal, in contrast to other cases, where it is thought to be trigonal bipyramidal. Making a distinction between potentially existing structures of transition states complicates appropriate selection of a given mechanism, since it often leaves out of consideration, whether the given structure represents a local minimum or a maximum on the reaction energy profile. At present it is not possible to collect direct experimental evidence for the existence of a given structure in the transition state, since its lifetime is practically zero. Quantum mechanical calculations can provide concrete models, like in Figure 6; however, these may not reproduce experimental data sufficiently accurately, and therefore conclusions on such basis are often questioned. York and coworkers studied the uncatalyzed conversion of PEP to phosphonopyruvate and they have found that due to the large negative charge located at the active site, solvation has a crucial effect on the reaction pathway [71].

**Figure 8:**
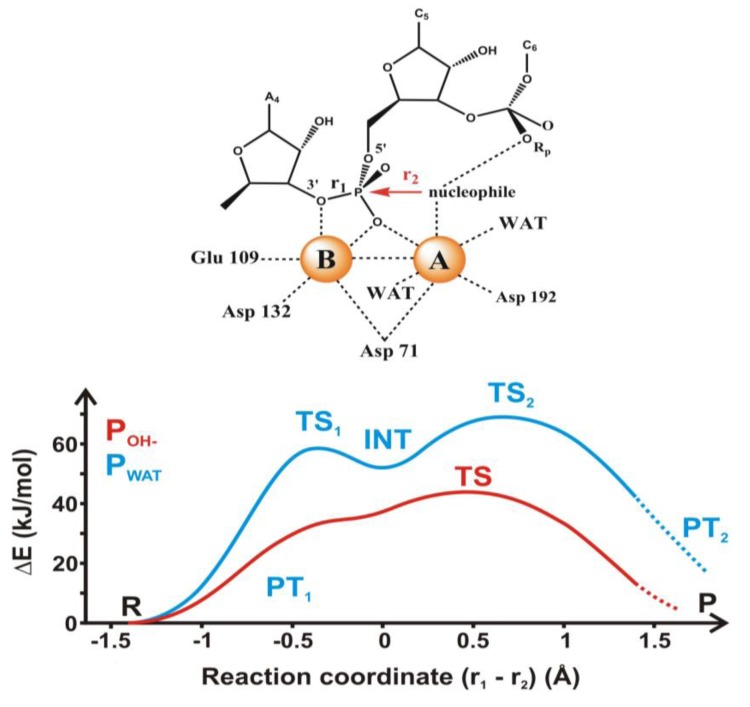
Two possible reaction routes for the hydrolysis of ribonuclease H. Top: schematic structure of the active site, bottom: blue line: attack by a water molecule, red line: attack by a hydroxyl group (figure drawn on the basis of Figure 3 of Ref. 72).

Two recent sophisticated quantum mechanical studies indicate that in ribonuclease H the phosphodiester cleavage occurs via an associative mechanism [72,73]. These calculations indicate that as the reaction approaches the barrier, one of the protons of the attacking water molecule transfers to one of the oxygen atoms of the phosphate group and a penta-coordinated phosphorane intermediate is formed. The calculated energy barrier is consistent with the experimental rate found for the human enzyme. De Vivo and coworkers made a distinction between the attack of a neutral water molecule and a negatively charged hydroxyl group [72]. They have found that in both cases an in-line S_N_2-like nucleophilic attack takes place on the central phosphorus atom. This results in an associative mechanism with phosphorane-like transition states, in agreement with crystal structures of transition- state analogues [74]. It is interesting that the attack of a water molecule leads to the formation of a meta-stable pentavalent phosphorane intermediate, as depicted in Figure 8. This is a unique characteristic of the energy profile of the reaction, which has not been observed in previous computational studies. Like in e.g., xylose isomerase (see below), one or more structural water molecules near the active site may facilitate the reaction [75,76].

Warshel and coworkers [77] compared the associative and dissociative mechanisms of phosphate monoester hydrolysis on the example of the methyl phosphate dianion and the methyl pyrophosphate trianion in aqueous solution. They have found that, in good agreement with experimental findings, both associative and dissociative transition states have near-zero entropies of activation. This means that near-zero activation entropy is not indicative of a dissociative pathway, as supposed earlier.

### 4.3. Long-Range Electron Transfer in Heme Peroxidases

**Scheme 1:**
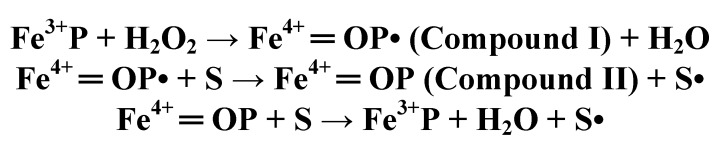
Reaction mechanism of heme peroxidases. P is the porphyrin group of the enzyme whose heme iron is indicated, S is the substrate.

**Figure 9:**
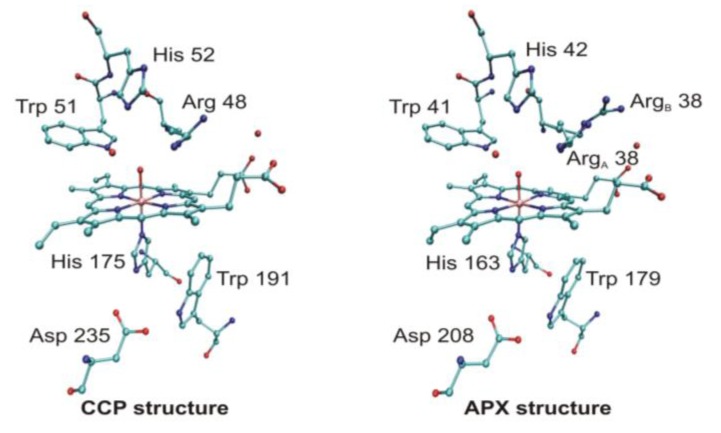
Schematic active-site models of cytochrome *c* peroxidase (CCP) (**left**) and ascorbate peroxidase (APX) (**right**). Top: distal position, bottom: proximal position, a separated red dot represents a water molecule (figure drawn on the basis of the crystal structures 1ZBZ and 2XI6).

Peroxidases oxidize a variety of substrates by reacting with hydrogen peroxide. In most cases the typical substrates are small aromatic molecules, however, in case of cytochrome *c* peroxidase (CCP) a protein, cytochrome *c*, is the redox partner. The overall reaction mechanism is given in Scheme 1. Several peroxidase structures were determined by X-ray diffraction (e.g., yeast cytochrome *c* peroxidase [78], lignin peroxidase (LIP) [79], pea cytosolic ascorbate peroxidase (APX) [80], and horseradish peroxidase (HRP) [81]. On this basis it is possible to construct a model of the active site (see Figure 9). A histidine and an aspartic acid side chain are present in the proximal heme pocket in all these structures, whereas the proximal tryptophan is only present in CCP and APX. 

**Figure 10:**
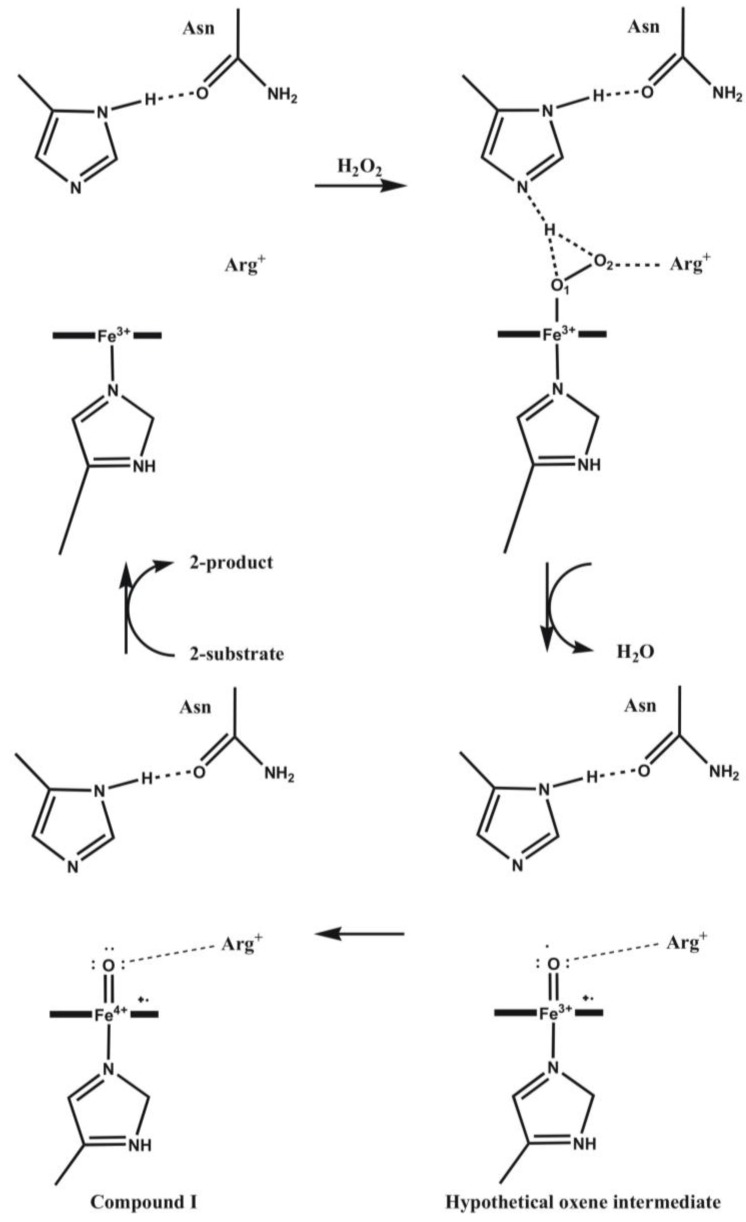
Catalytic mechanism of the formation of Compound I. The distal His assists in removing a proton from the incoming peroxide and delivering it to the peroxide O_2_ atom.

In the first step of the catalytic process (cf. Scheme 1 and Figure 10) the peroxide removes two electrons from the enzyme and the so-called Compound I is formed. During this process the peroxide O–O bond is broken, water is produced and the second oxygen atom of the peroxide remains coordinated to the metal, which loses one electron to give an oxy-ferryl intermediate (Fe^4+^ = O). A second electron is removed from the heme providing a cation radical. In the next step, P• is reduced and a substrate radical, S• is formed. Finally, in the last step Compound II is reduced by a second substrate molecule.

Discussing the mechanism at the computational level an interesting problem arises. APX shares 33% global sequence identity with CCP and has a very similar active site structure [82]. Despite very similar protein structures, APX and CCP stabilize different radical species during enzyme turnover. At variance with CCP, in APX the free electron is shifted to the porphyrin ring of the active site during reaction. This difference is thought to contribute to the different substrate specificities of the two proteins. Earlier, we suggested, on the basis of *ab inito,* minimal basis set molecular orbital and Poisson-Boltzmann electrostatic potential calculations that both in APX and CCP proton transfer involving the proximal histidine (His163 or His175), the radical-forming tryptophan (Trp179 and Trp191), and a nearby aspartic acid (Asp208 and Asp235) side chains may control the location of the radical state [83]. Both molecular orbital and electrostatic potential calculations suggest that the spin distribution depends on the protonation state of the proximal His...Asp...Trp triad. If the transferable proton is shifted from the Trp side chain to Asp, the free electron is localized on the indole group, while if it remains on Trp the unpaired electron transfers to the heme group. Therefore, in Compound I of CCP Trp is supposed to be deprotonated, while in case of APX it is supposed to be protonated and neutral. Protonation state of the side chains is influenced by the electrostatic effect of the protein environment, which differs in these enzymes, especially in the immediate vicinity of the Asp side chain. 

The above hypothesis is not supported by some other calculations. Warshel and coworkers applied the empirical Protein Dipoles Langevin Dipoles method and found that the electrostatic effect of the protein environment of CCP stabilizes the tryptophan cation radical by 330 mV relative to that in APX. They proposed that mainly the cation binding site contributes to radical stabilization, but is not the sole cause [84]. The study by Siegbahn and coworkers [85] supports the mechanism, which has been proposed on the basis of X-ray diffraction studies. They have found that if the proximal His-Asp-Trp triad of both enzymes is included in the computational model the free electron of the cationic radical is shared between the porphyrin ring and the protonated histidine side chain. The difference in ionization potential between tryptophan and porphyrin ring in the oxyferryl form of APX and CCP seems to be small. This strengthens the statement that location of the free electron depends on subtle differences of the protein environment in both enzymes. It has been shown that location of the protein radical in APX is most probably Trp179 [86]. Quantum mechanical optimization of the crystal structure of the active site in CCP show that the geometric differences of the two states are quite small and it cannot be decided where the proton is in the crystal structure [87]. QM/MM calculations indicate that the proton is located on the His ligand in all states in the reaction mechanism. According to de Visser [88] the quartet-doublet energy splitting is strongly dependent on local perturbations. Even a point charge far away from the tryptophan radical can transfer the system from a mixed porphyrin-tryptophan radical into a pure porphyrin or tryptophan radical. These perturbations cause varying quartet-doublet energy splitting, which eventually may influence reactivity. Calculations by Mulholland and coworkers [89] reproduced the observed difference in electronic structure, and called the attention to the subtle electrostatic effects which may affect the ionization state of both the tryptophan and porphyrin groups. Their calculations did not support the deprotonation of the tryptophan group, or protonation of the oxoferryl oxygen atom. The remarkable difference in electronic structure between the compound I intermediates in CCP and APX seems to be due to differences in the electrostatic potential around the key groups in the two enzymes. 

On the basis of their QM/MM calculations Thiel and coworkers concluded that in another heme peroxidase, horseradish peroxidase the proximal ligand is imidazole and not imidazolate [90].Similar conclusions were drawn by Jensen and Ryde for reduced models of heme proteins [91]. A recent review on QM/MM calculations for heme proteins is also available [92], while the catalase activity of heme peroxidases is treated by Vlasitsa *et al*. [93]. Harris and Loew [94] performed density functional calculations and showed that the imidazolate/Asp-H and imidazole/Asp**^–^** tautomers are very close in energy (the difference is 4 kJ/mol), but only the first representation reproduces the observed shift in the Soret band of Compound I in horseradish peroxidase as compared to chloroperoxidase. 

### 4.4. Cytochrome P450 Enzymes

Cytochrome P450 enzymes (P450s) form a huge superfamily of thiolate-ligated heme enzymes with more than 10,000 members sequenced up-to date. They can be found in almost all living organisms, in bacteria and archaea, in fungi, plants and animals indicating the ancient nature of these enzymes. The great variety of P450s can be attributed to their vital role: they defend the organisms from xenobiotics by oxidizing them to products that can be excreted more easily. As the structure and properties of xenobiotics may differ almost indefinitely, during evolution a very large number of P450s have evolved [95], in some cases acquiring new functions as well. P450s also play a vital role in hormone biosynthesis in mammals, e.g., in man they are responsible for the aromatization of androstenedione and testosterone to estrone and estradiol [96]. In the following we present three case studies, which may shed light on some interesting issues related to P450s. 

#### 4.4.1. Is the Oxidizing Power of Active Species of Various P450 Isoforms the Same?

**Figure 11:**
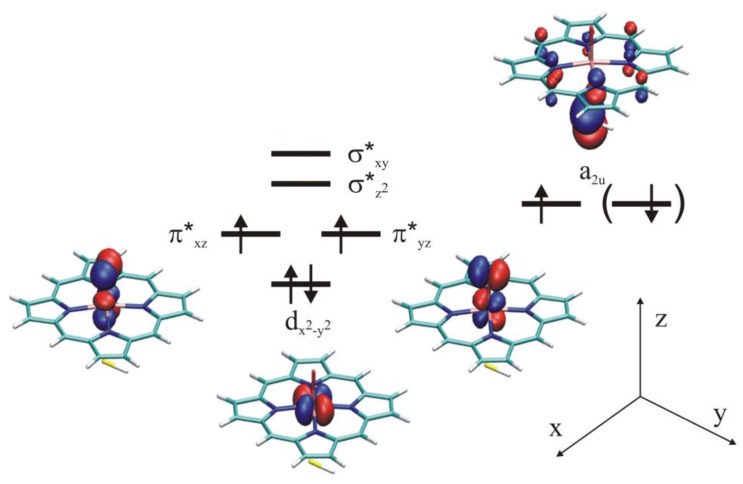
Singly occupied molecular orbitals of Compound I.

The catalytic cycle of P450s consists of many electron or proton transfer steps [97], which leads to the formation of Compound I, the ultimate oxidizing species carrying out the oxidation reaction. The species is very reactive and elusive that has only been recently observed [98]. Compound I has low-lying quartet and doublet spin states, which differ in energy by less than 4 kJ/mol. In both states there are two electrons in the Fe**–**O π* antibonding orbitals which can be coupled via an anti- or ferromagnetic scheme to the porphyrin a_2u_ orbital with significant contribution from the sulfur *p* orbital giving rise to the doublet and quartet states. (Figure 11.) As several P450 isoforms are responsible for the metabolism of drugs taken by man several crucial questions have been raised: (1) does the reactivity of the different isoforms depend on the oxidizing power of the isoform; (2) is the oxidizing power of Compound I of the different isoforms very similar? As the active site of the different isoforms shows great variability it may tune the reactivity of the oxidizing species and influence the regioselectivity of the enzyme [99,100], which has been called “chameleon” behavior [101]. Therefore, a systematic study involving three human P450 isoforms (P450 2D6, P450 2C9, P450 3A4) and a bacterial enzyme (P450_cam_) has been conducted in the presence and absence of substrate in the active site in order to reveal the similarities and differences between the active species of these isoforms [102]. Dextromethorphan was used as a substrate of P450 2D6 and P450 3A4, warfarin as the substrate of P450 2C9, and propene as the substrate of P450_cam_. 5ns long molecular dynamics simulations were run on the ligand-free and ligand bound enzymes and every 200 ps a snapshot has been taken (altogether 26 for each system) that was subjected to QM/MM optimization, in the quartet spin state. After the QM/MM optimization of each snapshot, the oxo group bonded to the iron was deleted and a single point calculation was carried out to estimate the energy of the resting state of the enzyme. 

**Figure 12:**
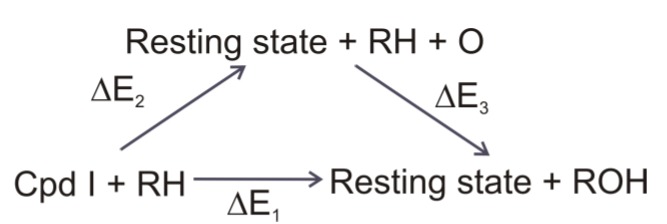
Thermodynamic cycle showing the relationship between ΔE_1_, ΔE_2_, (Fe-O bond enthalpy) and ΔE_3_.

The thermodynamic cycle, shown in Figure 12, depicts the importance of the Fe**–**O bond enthalpy (ΔE_2_) calculated in the study. ΔE_1_ includes the energy of oxidation of the substrate by Compound I to form the product and the resting state of the heme. Its value is dependent on the substrate, and could be expressed as ΔE_1_ = ΔE_2_ + ΔE_3_, where ΔE_2_ is the energy required to break the Fe**–**O bond in Compound I, and ΔE_3_ is the energy released upon addition of the oxygen atom to the substrate to yield the product. ΔE_2_ is independent of the substrate, and smaller values correspond to a more reactive and oxidizing state of Compound I. In our study ΔE_2_ was estimated as ΔE_2_ = E_QM_(O) + E_QM_(Fe) − E_QM_(Fe*) + E_QM/MM_(Fe*) − E_QM/MM_(FeO), where E_QM_(O) is the energy of oxygen *in vacuo* in the triplet state, E_QM_(Fe) − E_QM_(Fe*) accounts for the relaxation energy of the resting state of the heme complex compared to the structure in Compound I and for the energy difference between the sextet and doublet states of the resting state (103 kJ/mol). E_QM/MM_(Fe*) was obtained by a single-point calculation on the QM/MM optimized geometry of Compound I by deleting the oxygen atom from it E_QM/MM_(FeO) is the QM/MM energy of Compound I. The last two quantities were calculated for each snapshot and averaged over each system. 

**Figure 13:**
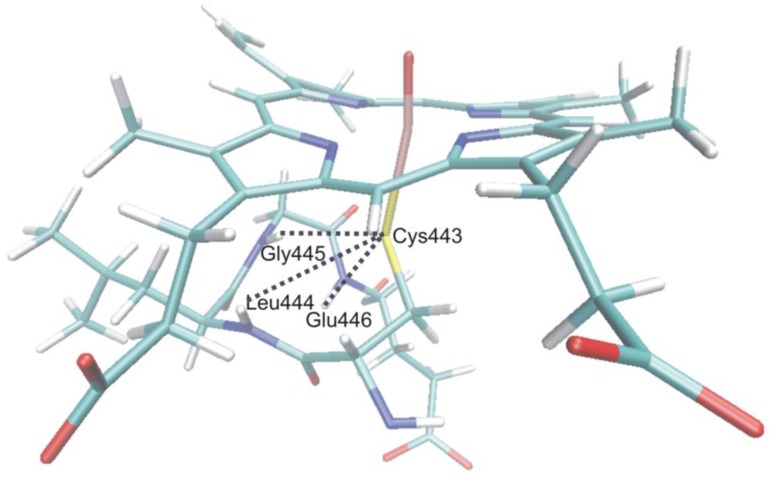
Quantum mechanical/molecular mechanical (QM/MM) optimized snapshot of Compound I with residues hydrogen-bonded to the axial cysteinate in P450 2D6.

In order to assess the electronic configuration of the oxidizing species, structural data (especially the Fe**–**S bond length), and charge and spin density data (on the Fe, O, S atoms and the porphyrin ring) have been collected and compared. All these data indicated that Compound I of the P450s is very similar in the studied human isoforms, as variation of the data for the different snapshots taken for a given isoform was larger than the variation between isoforms. However, the bacterial P450_cam_ isoform showed an increased spin density on sulfur compared to the human isoforms. It has been suggested that the different hydrogen bonding environment of the sulfur atom could be the main reason behind it. In P450s the axial cysteinate ligand of the heme is hydrogen bonded to the amide groups of the three consecutive amino acids of the protein chain as depicted for P450 2D6 in Figure 13, and one of the hydrogen bonds to sulfur was found to be much stronger in P450_cam_ than in the human isoforms. Upon substrate binding a slight decrease in the spin density of sulfur was observed in all cases, which was attributed to the displacement of water molecules from the active site, thereby changing its polarity. These results imply that the properties of Compound I are sensitive to changes in the hydrogen bonding environment and also to polarization effects exerted by the active site. 

The Fe**–**O bond enthalpy does not show great variability over the various isoforms either, as its variation is on the order of the same magnitude for the different snapshots of a given isoform as those between the various isoforms. This result suggests a similar oxidizing power for the different isoforms, which is slightly lowered in the presence of the substrate in the active site. This latter effect is most likely due to the fact that the substrate expels the water out of the active site and decreases the number of hydrogen bonds to the ferryl oxygen. As a consequence the spin density on oxygen increases and the charge associated to it decreases. It was also shown that structures with largest spin density on oxygen have the lowest Fe**–**O bond enthalpy. 

The overall conclusion of this study is that active species of the studied P450 isoforms exhibited very similar properties; therefore, it is reasonable to assume that the reactivity of Compounds I of different isoforms will be very similar. However, it was also shown that the electronic structure of Compound I varies with thermal fluctuations, therefore conclusions drawn on a single QM/MM optimized snapshot may not be necessarily reliable and it could be more appropriate to average the data over a number of structures. 

#### 4.4.2. Metabolism of Dextromethorphan by P450 2D6: What Drives the Regioselectivity of the Enzymes?

**Figure 14:**
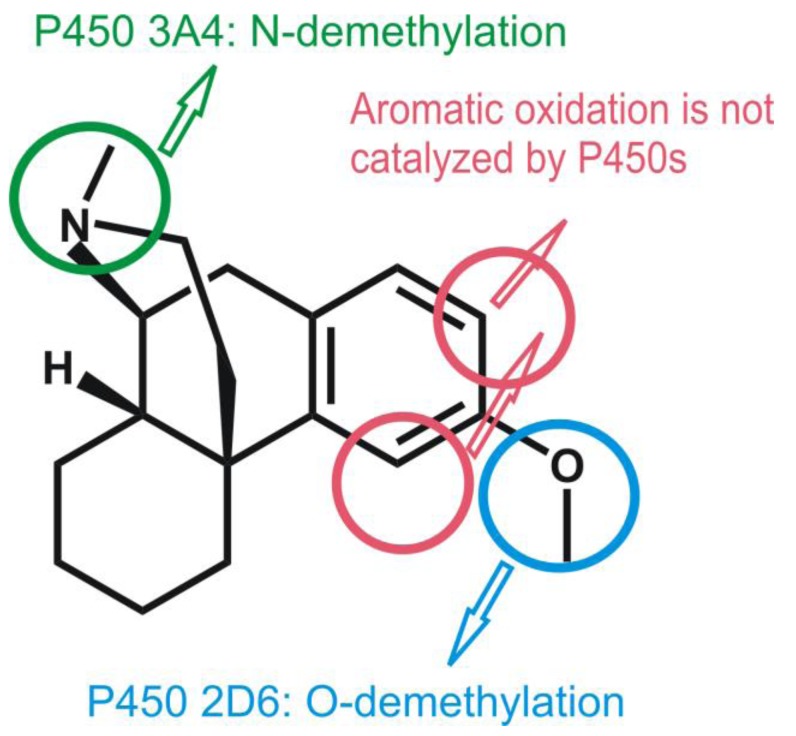
Metabolic routes of dextromethorphan in man as indicated by arrows.

Most P450s are promiscuous enzymes: a substrate molecule may be metabolized by various P450 isoforms leading to different products. A good example of this is the case of dextromethorphan, a common antitussive compound, which is even frequently used in various experimental studies of the P450 2D6 enzyme. In the human body, dextromethorphan is metabolized by P450 3A4 yielding an N-demethylated product (see Figure 14), while O-demethylation is catalyzed by P450 2D6 [103]. In contrast, the aromatic ring of this compound is not oxidized, despite the fact that aromatic oxidation of other aromatic ethers have been observed in rat [104] and rabbit [105]. The interesting metabolic properties of dextromethorphan make it a good candidate for the computational study of the factors influencing the regioselectivity of P450 isoforms. Earlier computation studies showed that in general the barrier of N-demethylation is lower than that of O-demethylation [106], therefore it is not astonishing that P450 3A4, which has a large active site capable of accommodating two substrate molecules and in which the substrate may be oriented in any position catalyzes the thermodynamically favored N-demethylation. However, it is more surprising that P450 2D6 only catalyzes the O-demethylation despite that fact that the aromatic ring is located close to the heme iron as well. 

**Figure 15:**
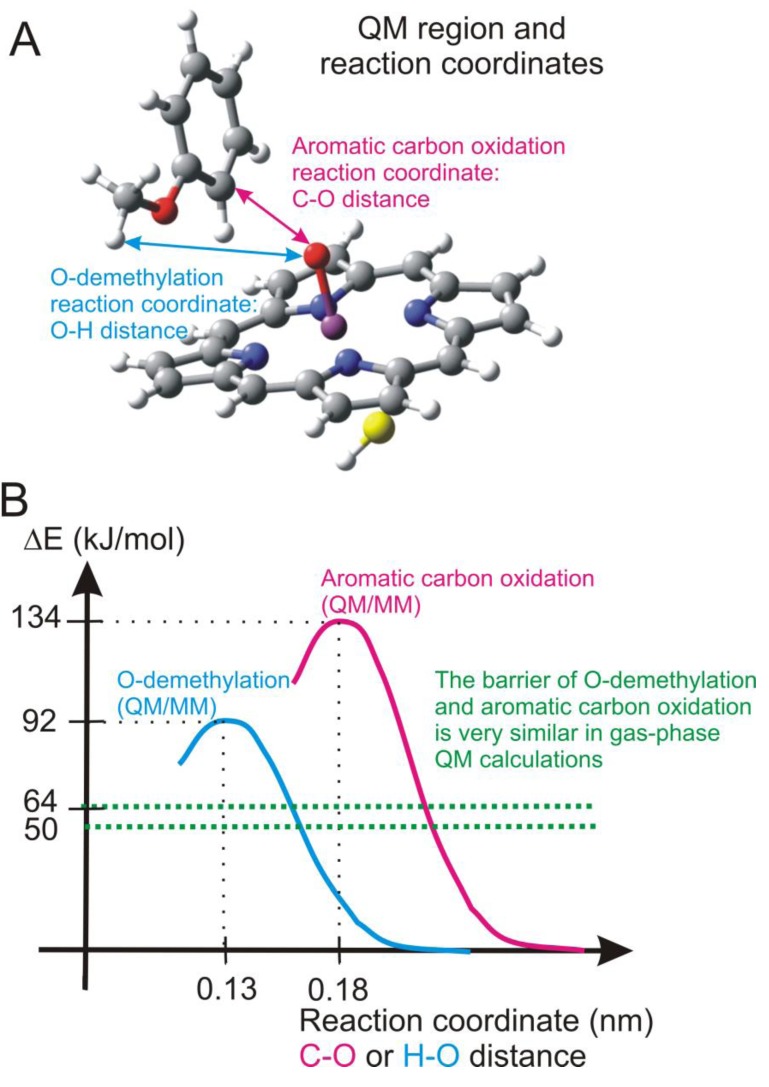
(**a**) QM region used in the calculations (**b**) Barriers of O-demethylation and aromatic carbon oxidation obtained from quantum mechanical and QM/MM calculations.

In order to clarify this problem, we conducted a study to investigate the factors influencing the regioselectivity of P450 2D6 [107]. First we carried out gas-phase quantum mechanical calculations on a model system (shown in Figure 15) to get an idea about the relative energy barriers of hydrogen abstraction from the methoxy group. This is the rate-determining step of O-demethylation, and of aromatic oxidation. Both reactions have a lower barrier in the doublet spin state than in the quartet spin state, 50.2 kJ/mol for hydrogen abstraction and 64.0 kJ/mol for aromatic oxidation. Although the barrier for O-demethylation is lower, the difference between the barriers of the two reaction channels does not explain why only O-demethylation is observed. 

The above result indicates that the regioselectivity of P450 2D6 might be also modified by its active site architecture. For this reason we turned our attention towards docking, molecular dynamics and QM/MM methods which are capable of taking into account the enzymatic structure. There are two important pharmacophores of the ligands of P450 2D6: all of them contain basic nitrogen and an aromatic ring [108]. Therefore the N-protonated form of dextromethorphan was used throughout together with the crystallographic structure (PDB code 2F9Q) [109] of P450 2D6. 

First, the ligand was docked into the active site of P450 2D6 in five different orientations, two of which seemed to be allowing for the O-demethylation reaction. In one of these two positions the protonated nitrogen of dextromethorphan interacts with the acidic Glu216 residue, and in the other one with Asp301, both of which have been implicated as important for catalysis [110]. In both structures the aromatic ring of the ligand participates in a π**–**π interaction with Phe120 whose mutation also leads to altered regioselectivity [111]. The structure in which interaction with Glu-216 was observed was chosen for further MD simulations and QM/MM calculations. During the course of a 2ns MD simulation, the docked structure maintained its major interactions with the active site residues, and it was observed that both the methoxy group and the aromatic ring of dextromethorphan remained in the close proximity of the heme iron during the whole time span of the simulation, indicating the possibility to be oxidized, in contrast to the fact that experimentally only the former one is known to occur. 

**Figure 16:**
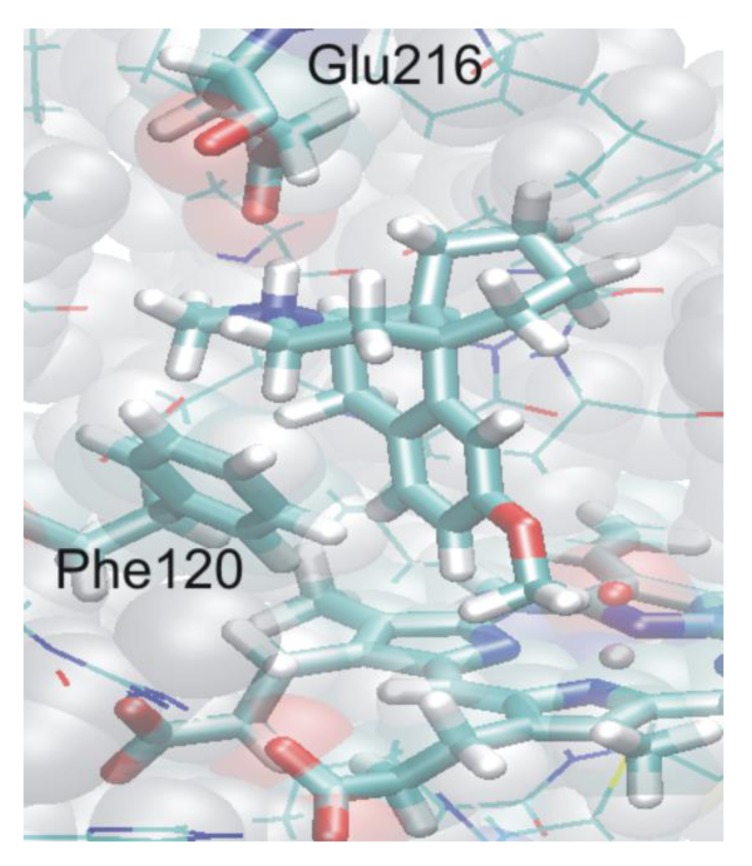
Active site of P450 2D6 with dextromethorphan. The movement of dextromethorphan in the active site is hindered by its salt bridge to Glu216 and by the steric hindrance of the bulky amino acid side-chains.

As neither gas-phase quantum mechanical calculations nor docking alone could explain the experimental regioselectivity, it seemed to be essential to combine in the calculations the accuracy of quantum mechanical calculations with the steric restraints exerted by the protein structure in the frame of QM/MM calculations. The trajectory of the MD run was thoroughly analyzed and 3–3 suitable starting structures were chosen to model the two reaction channels. The QM region in the QM/MM calculations consisted of the same atoms as in the quantum mechanical calculations (Figure 15), but the axial thiolate ligand was modeled by an SCH_3_^-^ moiety. Dangling bonds were saturated by hydrogen-type link atoms. Using adiabatic mapping, which gives an estimate of the enthalpy component of the Gibbs-free energy of the reaction, we obtained 92 kJ/mol for the barrier of hydrogen abstraction (O-demethylation) and 134 kJ/mol for the barrier of aromatic carbon oxidation. The difference between the activation energies of the two reaction channels increased to about 40 kJ/mol in contrast to the 14 kJ/mol calculated for the gas-phase (see the green dotted lines in Figure 15). The reason for the great increase of the barrier of the aromatic carbon oxidation is that the active site of P450 2D6 is relatively tight and the movement of dextromethorphan is hindered by several non-polar active site residues, e.g., Phe120, Ala305, Val308 (see Figure 16). Therefore the favorable transition state structure for aromatic oxidation cannot be formed in the active site, and its barrier considerably increases. The most important conclusion of the study was that gas-phase calculations or docking studies alone or even when they are combined are not enough to predict the regioselectivity of the P450 2D6 enzyme and more sophisticated models are needed. It was suggested that the metabolite predicting algorithms might be improved by docking the approximate transition state structures into the active site of the enzyme and assessing the feasibility of its formation [101]. 

For a comprehensive study of the oxidation of further three pharmacologically important molecules see Lonsdale *et al*. [112]. 

#### 4.4.3. Reactivity of the P450_nor_ Enzyme

Nitric oxide reductase (P450_nor_) is a very special member of the superfamily of P450s. Instead of the most generally catalyzed oxidation reactions of P450s, it catalyzes the reduction of nitric oxide to dinitrogen oxide and for its proper functioning it does not require a redox mediator, but contains its own NADH binding site [113]. P450_nor_ is a soluble protein found in the cytosol of the fungus *Fusarium oxysporum,* a denitrifying organism, which can generate N_2_O from NO_3_^-^ ions in three main steps using three different enzymes. The last step of the conversion is nitric oxide reduction to N_2_O by nitric oxide reductase. Since N_2_O has a 300 times larger greenhouse effect than carbon dioxide [114], it should be taken into account in chemically fertilized nitrate abundant area [115]. Nitric oxide plays an important role in mammals for instance as a neurotransmitter or as part of the immune response against pathogens [116]. *Histoplasma capsulatum* is a human pathogen, which can avoid damage using the Nor1p enzyme, which shows great similarity to P450_nor_ (61% sequence identity, 79% similarity) [117], and a similar mode of action can be assumed for converting NO. 

**Scheme 2:**
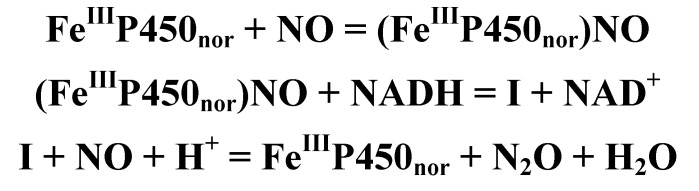
Conversion reaction catalyzed by P450_nor_.

The conversion catalyzed by P450_nor_ takes place in three main steps (see Scheme 2). The first step is the binding of nitric oxide in the active site of P450_nor_, which is followed by hydride transfer to (Fe^III^P450_nor_)NO from NADH to form the intermediate (**I** in Scheme 2.). After the binding of a second molecule of nitric oxide, the final product, N_2_O, is generated and the resting state of the enzyme is restored. Several suggestions have been put forward for the structure for the intermediate based on experimental and theoretical investigations [118,119,120,121,122,123]. According to the most accepted and experimentally proven mechanism direct hydride transfer occurs from the NADH co-factor to the oxygen or nitrogen atom of nitric oxide. DFT calculations performed by Lehnert *et al*. suggested that the hydride is most likely transferred to the nitrogen atom of nitric oxide in the key step, and intermediate (**I**) is the doubly protonated [Fe(Porph)(CH_3_S)(NHOH)] species [123]. Based on the calculated relative energies at the B3LYP/LanL2DZ* and BP86/TZVP levels the [Fe(P)(CH_3_S)(NHO)]^-^ species is 109.7 kJ/mol more stable than the [Fe(P)(CH_3_S)(NOH)]^-^ species, and the protonation of [Fe(P)(CH_3_S)(NHO)]^-^ is a slightly exothermic process (ΔG = −36.0 kJ/mol).

In the previous sections it was shown that steric and electronic factors significantly influence the reactivity of enzymes, and that QM/MM calculations may provide a better description of enzymatic processes than gas phase calculations alone. Two QM/MM studies addressing the reactivity of P450_nor_ were published recently, however dealing with partially different aspects of its reactivity. Our study focused on two key questions, (1) to which atom of nitric oxide will the hydride be transferred, and (2) how does the origin (docked or co-crystallized) influence the outcome of QM/MM calculations [124]. To answer the questions we used quantum mechanical and QM/MM methods, and based on experimental findings we assumed that the spin multiplicity of the system is singlet [125,126] and applied a closed-shell formalism in the calculations. 

The results suggest that in the active site pocket of P450_nor_ hydride transfer from NADH occurs to the nitrogen end of nitric oxide. In this respect all of our calculations (quantum mechanical or QM/MM) provided the same result, but the barriers were significantly higher in the QM/MM calculations. For instance the calculated barrier for hydride transfer from NADH to the nitrogen atom of nitric oxide is 37.3 kJ/mol in quantum mechanical and 113.5 kJ/mol in QM/MM calculations including zero-point energy correction in contrast to the 139.0 kJ/mol (quantum mechanical) and 161.2 kJ/mol (QM/MM) values obtained for the hydride transfer to the oxygen end of nitric oxide. The obtained high QM/MM barriers were most likely due to the incomplete sampling of initial complexes, and the chosen complexes did not provide an ideal starting structure for the reaction to occur. Nevertheless, the relative energy of the barriers convincingly showed that the hydride transfer will occur to the nitrogen of nitric oxide. 

**Figure 17:**
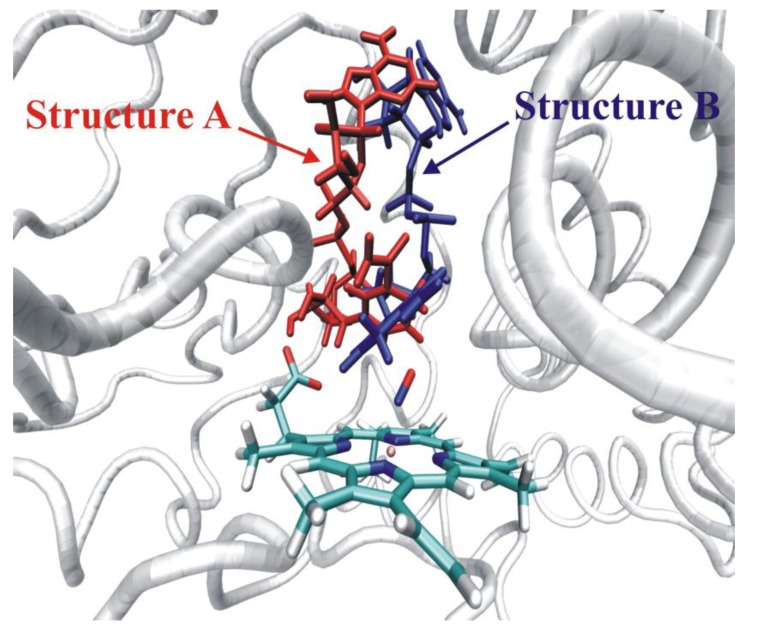
The position of the NADH ligand in the docked structure (structure **A**) and in the crystal structure (structure **B**).

Comparing QM/MM calculations on two different structures of NADH bound P450_nor_ brought also interesting results. Two structures were used. In structure **A** NADH has been docked to the active site of the NO-bound P450_nor_ enzyme (Protein Data Bank code: 1CL6) [127], while structure **B** was obtained from the crystal structure of P450_nor_ in complex with NAAD (nicotinic acid adenine dinucleotide), an analogue of NADH (Protein Data Bank code: 1XQD) [128]. In the latter structure nitric oxide was manually inserted. The position of the ligand and its interaction with active site residues was significantly different in the two models (see Figure 17). In structure **A** the pro-S hydrogen, while in structure **B** the pro-R hydrogen atom of NADH could be transferred as hydride to nitric oxide. We obtained significantly lower barriers using structure **B**, in accordance with the results of experimental kinetic isotope effect measurements, which also suggested the transfer of the pro-R hydrogen [121]. Therefore our results imply that the selection of docked structures as models should be avoided, since upon ligand binding a major rearrangement of the active site may take place. Unfortunately, present docking methods cannot take these rearrangements into account, and therefore the obtained results on the regioselectivity of the enzyme may contradict to experiments, furthermore, barrier heights may also be considerably influenced. 

**Figure 18:**
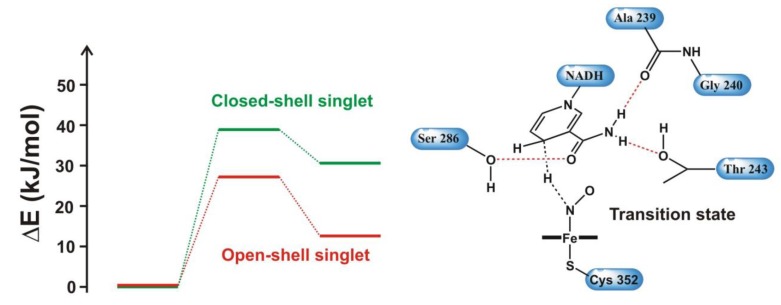
Energy profiles for closed-shell and open-shell singlet pathways for hydride transfer in the P450_nor_ and the schematic structure of the transition state.

In another QM/MM study hydride shift directed to the nitrogen atom of nitric oxide was investigated [129]. This study resulted in considerably lower energy barriers: 38.9 kJ/mol for a closed-shell system at the DFT(RIJCOSX-B3LYP)/MM(OPLS-AA) level including zero-point energy correction. The lower barrier could be explained by the presence of a stabilizing hydrogen bond interaction between the amide group of the NADH molecule and the Ser286, Thr243 and Ala239 residues (see Figure 18), which are responsible for stabilization of the transition state. These were absent in models used in the work discussed above. When the authors applied an unrestricted formalism to obtain the wave function of the broken-symmetry singlet state, the calculated barrier further dropped to 27.2 kJ/mol, and showed that that the open-shell singlet state of the [Fe(Porph)(Met)(NHO)]^-^ species is by about 16.7 kJ/mol more stable than the closed-shell one. The study also suggested that the N**–**N bond of N_2_O is formed in a spin-recoupling process, followed by the release and spontaneous decomposition of the N_2_O_2_H_2_ ligand to N_2_O and H_2_O. 

### 4.5. Xylose Isomerase

**Figure 19:**
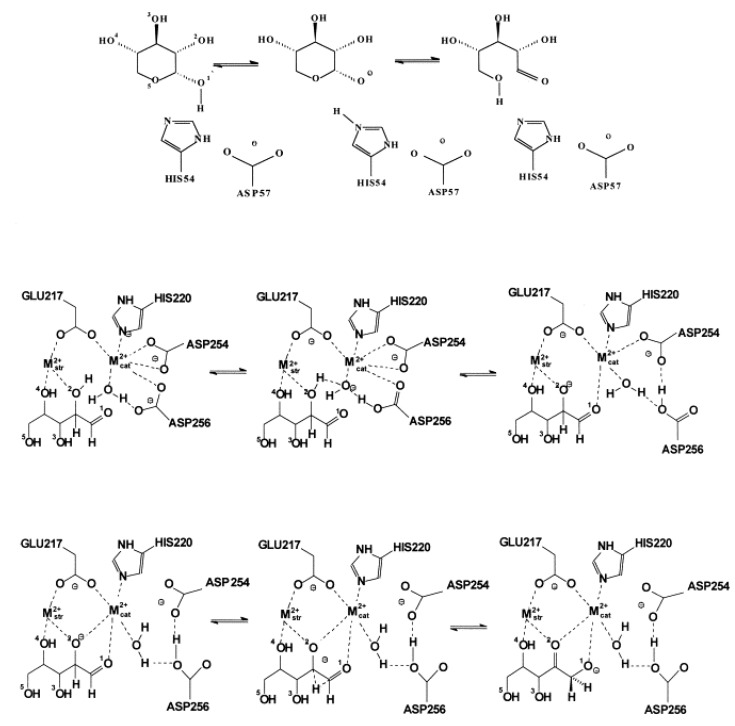
Catalytic mechanism of xylose isomerase**.** Top: ring opening, middle: substrate deprotonation, bottom: hydride shift.

D-Xylose isomerase catalyzes the conversion of D-xylose to D-xylulose and the transformation of other sugars from aldose to ketose. It is widely used in industry for the conversion of D-glucose to D-fructose. The enzyme is activated by divalent metal cations (Mg^2+^, Co^2+^ or Mn^2+^), whereas other cations (e.g., Ca^2+^, Ba^2+^ and Al^3+^) inhibit the reaction [130]. The 3D structures of xylose isomerases isolated from different species have been extensively investigated by X-ray diffraction techniques [131,132,133]. On this basis the following mechanism of action has been proposed (see Figure 19 and Ref. 5). Binding the ring form of the sugar substrate to the structural metal ion is followed by a ring-opening step in which the hydrogen atom of the hydroxyl group on C1 is transferred to O5. This step is accompanied by the formation of a (**–** **+** **–**) charge distribution, involving the substrate hydroxide; His54 and Asp57, similar to those found in serine proteases (see Section 4.1). In the extended conformation, O2 and O4 of the sugar substrate are bound to the structural metal ion (M_str_), while the catalytic metal ion (M_cat_) is not in direct contact with the substrate. A structural water molecule coordinated to M_cat_ removes the OH2 proton of the substrate. Isomerization takes place via hydride-shift, in which the C2 hydrogen is transferred to C1 via an anionic transition state, assisted by M_cat_. This is the rate-limiting step of the reaction. 

Extended calculations have been done on the validity of the above mechanism by Hu, Shiu and Li [134]. Their results indicate that in the enzyme M_cat_ moves from one site to another, which is close to the substrate and stays there. M_cat_ acts as a Lewis acid, polarizes the substrate and catalyzes the hydride shift step. Calculations showed that Lys183 plays an important role in the isomerization reaction, because its protonated terminal ammonium group provides a proton to the carboxide ion of the substrate to form a hydroxyl group after the hydride shift step. If this side chain is dropped from the active-site, model proton transfer instead of hydride shift is found to be rate limiting [135]. The calculated activation energies for ring opening and hydride shift steps were found to be 42 and 71 kJ/mol. respectively. If the activating metal cation, Mg^2+^, was replaced by an inhibiting one, Zn^2+^, calculated activation energies increased to 63 and 138 kJ/mol, respectively.

**Figure 20:**
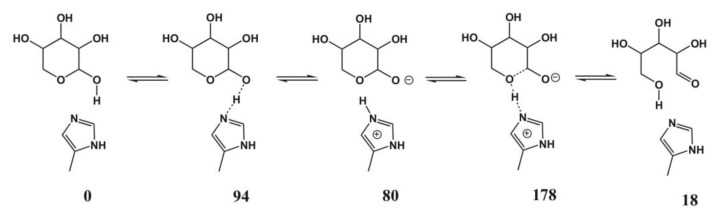
Initial, transition and final structures in the proton transfer steps of the ring opening reaction (relative energies are given in kJ/mol).

Our early calculations [136] indicated that the sugar ring opens only after the proton becomes shared between NE2 of His54 and O5 of the sugar. Ring opening is initiated by the approach of Asp57 towards His54 enhancing the basicity of the imidazole side chain of the latter. After the transition state has been reached, the movement in the opposite direction has been predicted by the calculations. The hypothesis that the charge-relay mechanism may be effective in catalysis, like in case of serine proteases, could be ruled out, because the electrostatic potential pattern provided by the protein environment and the bound metal cation (M_str_) stabilizes the ion-pair form of the Asp57**^–^**…NE2^+^…O1**^–^** triad. The negatively charged side chains of Asp57, as well as two bound structural water molecules assist in stabilizing the polar transition states. As it is seen in Figure 20, the activation energies of the proton transfer from O1 to NE2, as well as from NE2 to O5 become quite high if we do calculations on a small model, excluding Asp57 and the structural water molecules.

The anomeric specificity of the enzyme was also accounted for, since only the α-anomer is properly positioned for a proton transfer. Similarly, Lambeir *et al*. found that it is the His54 residue, which is responsible for the conformational specificity of xylose isomerases [137]. After abstracting a proton from the structural water by Asp256 the hydroxyl ion formed binds to the O2H moiety of the substrate, the structural water breaks the M_cat_…Asp256 link and a hydrogen-bond forms between them. 

A specific effect, hydrogen tunneling takes place in xylose isomerases. This is especially interesting, since it cannot be described by other methods; specific quantum mechanics is needed. Gao and coworkers combined molecular dynamics with quantum mechanics including consideration of hydrogen tunneling to calculate the reaction rate of the transformation of xylose catalyzed by xylose isomerase in the presence of two divalent magnesium cations. Their model includes more than 25,000 explicit atoms. The simulation confirms the essential features of a mechanism involving a rate-determining 1,2-hydride shift with prior and post proton transfers. They have found that inclusion of quantum mechanical vibrational energy is important for computing the free energy of activation, and quantum mechanical tunneling effects are essential for computing kinetic isotope effects. It is predicted that 85% of the hydride shift reaction proceeds by tunneling. The computed kinetic isotope effect was found to be in good agreement with experimental results. The molecular dynamics simulations reveal that proton and hydride transfer reactions are assisted by breathing motions of the mobile Mg^2+^ ion in the active site during the hydride transfer step [138,139].

Further studies have been reported on tunneling in various other enzymes, too. The reaction pathway for tryptamine oxidation by aromatic amine dehydrogenase was studied by Masgrau *et al*. [140]. It has been shown by combining experiment and computer simulation, that proton transfer occurs in a reaction, which is dominated by tunneling. In this enzyme system instead of long-range coupled motions tunneling is promoted by a short-range motion modulating the proton-acceptor distance. Hydrogen tunneling was studied in a model system that represents the active site of soybean lipoxygenase-1. Calculations showed that the tunneling process has a three-dimensional nature [141].

## 5. Conclusions

Enzyme reactions involve a large number of atoms, of which some, belonging to the active site, are crucial in understanding the process, while others are of less, however, not negligible importance. Owing to this special situation, special models and computational methods are needed for an adequate description. The active site can be modeled by small organic structures and some basic features of the reaction mechanisms can be elucidated by these. Calculations on this region must be at the quantum mechanical (Hartree-Fock, density functional or Empirical Valence Bond) level since bonds are broken and formed during the reaction, this needs a special treatment. More distant regions may be also of some importance, especially if the electrostatic field induced by them at the active site is strong. This is the case in point mutants where an ionizable side chain, which is close to the active site becomes charged or neutral by mutation. Those reactions, which involve a change in the polarity of the active site during transformation to the transition state, are strongly influenced, in most cases accelerated by the protein electrostatic field. In some cases subtle changes in the structure of the protein core may trigger essential changes in the mechanism. X-ray diffraction studies provide adequate models for the calculations.
